# Perceptions of Technology and Its Use for Therapeutic Application for Individuals With Hemiparesis: Findings From Adult and Pediatric Focus Groups

**DOI:** 10.2196/rehab.3484

**Published:** 2015-02-10

**Authors:** Melanie Y Lam, Sandy K Tatla, Keith R Lohse, Navid Shirzad, Alison M Hoens, Kimberly J Miller, Liisa Holsti, Naznin Virji-Babul, HF Machiel Van der Loos

**Affiliations:** ^1^Department of Human KineticsSaint Francis Xavier UniversityAntigonish, NSCanada; ^2^Department of Occupational Science and Occupational TherapyUniversity of British ColumbiaVancouver, BCCanada; ^3^Sunny Hill Health Centre for ChildrenVancouver, BCCanada; ^4^School of KinesiologyUniversity of British ColumbiaVancouver, BCCanada; ^5^Biomedical Engineering Graduate ProgramUniversity of British ColumbiaVancouver, BCCanada; ^6^Department of Physical TherapyUniversity of British ColumbiaVancouver, BCCanada; ^7^Child and Family Research InstituteVancouver, BCCanada; ^8^Department of Mechanical EngineeringUniversity of British ColumbiaVancouver, BCCanada

**Keywords:** cerebral palsy, stroke, hemiplegia, rehabilitation, gaming, social media, technology adoption, qualitative research

## Abstract

**Background:**

Digital technology is becoming an increasingly popular means of delivering meaningful therapy to individuals with neurological impairments. An understanding of clients’ technology use and their perspectives on incorporating technology into rehabilitation can provide researchers and designers with valuable information to inform development of technologies and technology-based rehabilitation programs.

**Objective:**

This study was designed to establish the current use and perceptions of gaming, social media, and robotics technologies for rehabilitative purposes from the perspective of adults and children with upper limb impairments to identify barriers and enablers to their adoption and use.

**Methods:**

We conducted three focus groups consisting of pediatric (n=7, mean age 11.0 years) and adult (n=8, mean age 60.8 years) participants with hemiparesis affecting their upper limb. We applied thematic analysis methods to the resulting data.

**Results:**

We identified three key themes: (1) clients’ use of technology in everyday life and rehabilitation, (2) barriers to use, and (3) enablers to therapy. Participants had limited exposure to technology for therapeutic purposes, but all acknowledged the potential benefits in providing motivation and interest for the performance of repetitive task practice. Adult participants requested efficacious, simple, and easy-to-use technology for rehabilitation with programs that could be individualized for them and expressed that they wanted these programs to provide a motivating means of repeated practice of therapeutic movements. In contrast, pediatric participants emphasized a desire for technology for rehabilitation that offered opportunities for social interaction and interactive games involving their whole body and not only their affected limb. Perceived safety and privacy were concerns for both groups.

**Conclusions:**

Our findings highlight that all participants were open to the integration of technology into rehabilitation. Adult participants were more pragmatically motivated by potential recovery gains, whereas pediatric participants were more intrinsically motivated by access to games.

## Introduction

Therapeutic exercises are an important component of a comprehensive rehabilitation program designed to improve strength, flexibility, mobility, and function of the affected limb(s) in individuals with hemiparesis, including those with cerebral palsy (CP) or stroke. Unfortunately, rehabilitation therapy tends to be terminated when clients shows no marked improvements within a set recovery time frame, or after a critical time period post stroke when rehabilitation is thought to no longer benefit them. As a consequence, any improvements in motor control and functional abilities that may have been acquired during therapy typically deteriorate over time [1]. The importance of maintaining an exercise regimen aimed at improving motor function once rehabilitation therapy ends has been demonstrated in studies examining individuals who showed continued motor function when engaging in repeated motor practice more than a year post stroke [2,3]. Indeed, as repetitive practice is an integral element in the functional retraining of individuals with stroke [4] and CP [5], home-based exercises are routinely prescribed to maintain or to improve functional gains obtained during inpatient and outpatient rehabilitation. Ultimately, these home exercise programs are intended to help individuals assume responsibility for the long-term management of their functional impairments. Despite the demonstrated benefits of these programs [6,7], adherence rates are suboptimal [8]. A number of factors have been identified as barriers to adherence to prescribed home-based exercise programs by individuals with CP and those who have suffered a stroke. These include personal (eg, motivation, time constraints), health (eg, fatigue, musculoskeletal problems), and environmental (eg, equipment, emotional/physical support) factors [9,10]. Studies have indicated that client motivation specifically has an influence on rehabilitation outcomes such that greater motivation is associated with a more favorable result [11-14]. Accordingly, researchers are now investigating methods of motivating clients to practice their therapeutic exercises outside of the clinical setting.

An increasingly popular motivational strategy is the use of computer gaming technologies to augment home exercise prescription (see [15] for a review). However, successful development, design, adoption, and use of these technologies hinge on understanding how clients think and feel about games, technology, and rehabilitation in the home setting [16]. Focus groups provide valuable insight from potential users in the early stages of product development because they identify the specific needs of the targeted user and can highlight to design teams the features of a product that could be problematic for users. For example, Demain et al [17] used the focus group technique to probe the views of individuals after stroke, health care professionals, and family caregivers on assistive technology and their perceptions of stroke upper-limb rehabilitation. Their study demonstrated that focus groups offer critical insight into the importance of including all stakeholders in the design process of assistive technology and its testing outside of the controlled setting of a laboratory. Other studies have also used focus group discussions to identify the suitability of commercial games as a rehabilitation tool for persons recovering from spinal cord injury, traumatic brain injury, and stroke [18] and to acquire feedback about home-based rehabilitation devices from children with cerebral palsy [19].

The purpose of this study was to explore potential users’ perspectives of technology for rehabilitation of the upper limb using information from focus group discussions. Specifically, the study aimed to determine to what extent the participants were currently using social media, computer games, and assistive devices, and their perceptions of these technologies in everyday life and rehabilitation. An additional aim was to explore the perceived relative advantages of incorporating these technologies into a client’s own rehabilitation for both adults and children with hemiparesis, and their caregivers, identifying barriers and enablers to use. Clinician perceptions of technology use for rehabilitation of the upper limb were investigated separately [20].

## Methods

### Participants

Persons with hemiparesis affecting their upper limb were invited to participate in the study by clinicians at two separate facilities within a publically funded child development center and a private clinic providing outpatient therapy for adults with neurological conditions. Two participant samples, an adult group and a pediatric group, were recruited (see Table 1 for the demographic and physical impairment information of all participants). The pediatric participants all attended school at their appropriate grade level. Note that one participant (P4: 12-year-old male with acute brain injury [ABI]) received additional behavior management support. The pediatric participants were required to have the ability to share verbal responses to focus group questions and to provide context-specific answers to those questions. Parents were present during the focus group discussion to help clarify responses when needed or to elaborate on a statement made by their child; however, they were not active participants in the discussion. The wife of one adult participant was present but did not participate in the focus group. All adult participants provided written informed consent. Informed child assent and parental consent were required from the pediatric group. Approval for this study was obtained from the UBC’s Research Ethics Board (REB #: H12-00220).

**Table 1:** Participant demographic information.

Focus group	Participant #	Gender, M/F	Age, years	School grade	Diagnosis	Caregiver present during session
1 (Pediatric)	1	M	13	8	CP, hearing impairment	Y
	2	M	6	1	CP	Y
1 (Pediatric)	3	M	11	5	CP	Y
	4	M	12	Not disclosed	ABI	Y
	5	M	16	11	CP	N
	6	F	8	3	CP, visual impairment	Y
	7	M	11	5	In utero stroke	Y
2 (Adults)	8	M	61	N/A	Stroke	N/A
	9	F	70	N/A	Stroke	N/A
	10	M	45	N/A	Stroke	N/A
	11	M	73	N/A	Stroke	N/A
	12	F	41	N/A	Stroke	N/A
	13	M	60	N/A	Stroke	N/A
	14	M	75	N/A	Stroke	N/A
	15	M	61	N/A	Stroke	N/A

### Data Collection

Focus groups were conducted as part of a larger project, Functional Engagement in Assisted Therapy through Exercise Robotics (FEATHERS) (intended to develop a home-based upper-limb rehabilitation platform), to obtain in-depth information from a group of participants representing potential users of the technology [21]. The semistructured focus groups took place in the facilities where the participants were recruited. At the beginning of each group, author MV introduced the FEATHERS project and described the development of rehabilitative technology, which might include gaming systems and/or robotic systems, as the background context for the focus group. During the focus group session, participants were led through a series of questions following a semistructured guide (see Multimedia Appendix 1 for samples of these questions) that was developed in conjunction with a team of qualitative research experts with experience in conducting focus groups. The focus group moderator (ST) is an occupational therapist with extensive experience in the management of individuals with neurological conditions. The moderator facilitated the discussion to allow the participants to enrich the conversation through interactions with each other and with project personnel. The questions probed the participants’ views of current therapy and use of technology, desirable features in a future technology designed to rehabilitate the upper limb, and perceived barriers to use of technologies.

The entire conversation was recorded and later transcribed verbatim by a research assistant. One of the research team members (ML) took detailed field notes to complement the transcription. The field notes reported participant characteristics, body language, the consistency between participant comments and observed behavior, and the overall mood of the discussion; they captured details that the audio recording could not. Transcripts identified participants and field personnel by number so that perceptions/contributions of each individual could be tracked anonymously throughout the conversation.

### Data Analysis

Anonymized transcriptions were given to four project personnel (authors KL, KM, ST, and NV) for coding based on thematic analysis [22,23]. The coders for the adult participant group were a cognitive neuroscientist with a specialization in motor learning and control (KL) and a physical therapist with 25 years of experience in the treatment of adults and children with neurological conditions (KM). Coders for the pediatric group were an occupational therapist and researcher in the field of pediatric neurorehabilitation (ST), and a physical therapist and professor in the Department of Physical Therapy with a specialization in developmental neuroscience (NV). Before the analysis, all coders wrote a statement of their personal background and potential biases/assumptions with respect to the general theme of the project. These explicit bias statements were used in later stages of the analysis (namely, reflecting on which codes were generated and how these codes were grouped into themes [24]). In the thematic analysis, the lowest level of information was individual codes (eg, “mirror-box”, “personal computer”) that were supported by multiple quotes or “extractions” from transcripts and supporting materials. These codes were then organized into categories (eg, “tools used”, “purposes for using”), subthemes (eg, “technology for rehabilitation”, “technology in the home”), and themes (eg, “client’s use of technology in everyday life and rehabilitation”).

Thematic analysis was conducted in five stages (based on recommendations by [22]). First, coders independently read the transcripts and the field notes to familiarize themselves with the data. Next, themes were generated based on the recurrence of ideas, topics, or words in the transcripts. Themes were generated to be semantic rather than latent in nature. That is, the coders attempted to minimize their own inferences, so that the themes were superficial and apparent in the text. Using an inductive approach, themes were generated based on the codes [25]. Coders reviewed the levels of their individual themes, subthemes, and categories prior to meeting together to generate consensus themes, which were refined using an iterative process. The goal of the coders when constructing themes was to provide a rich description of the full dataset. Finally, all coders met as a group led by a researcher with expertise in qualitative research (LH) to explore and refine the specifics of each theme.

## Results

Three focus groups (one group of adult participants and two groups of pediatric participants), for a total of 15 participants, were conducted between November 2012 and March 2013. The focus group data are presented as quotations from individuals. It is important to note that even though these quotes represent individual statements, there was considerable interaction between focus group participants that shaped these statements.

Three major themes emerged as being key to understanding participants’ perspectives of technology and its use for therapeutic rehabilitation: (1) clients’ use of technology in everyday life and rehabilitation, (2) barriers to use, and (3) enablers to therapy, which includes motivating factors and desirable features discussed by participants. Tables 2 and 3 summarize the features that participants identified as desirable for incorporation in gaming systems.

**Table 2:** Summary of main barriers to use and main enablers to therapy for adult focus group.

Features		Representative quotes
**Main barriers to use**		
	Cost-efficient	Context: the burden of out-of-pocket expenses for therapy beyond the number of funded rehabilitation treatments they can receive.
		Adult participant: …for people without money I don’t think that’s fair
	Assurance of therapeutic improvement	Context: how busy lives make it challenging to commit to a home-based rehabilitation program.
		Adult participant: We don’t have time, but if you said this is going to help you then we would do it…so you have to say I’m going to do this every day for fifteen minutes, say, or whatever. And if it works and someone like me, you see a difference, well it spurs you on, right?
**Main enablers to therapy**		
	Distinct exercises from those practiced in the clinic	Context: technology as motivation for rehabilitation by offering alternative exercises to those performed in the clinic.
		Adult participant: …it’s so boring to sit there and roll a towel up.
	Game-based therapy to generate results	Context: importance of repetition of exercises in stroke rehabilitation.
		Adult participant: In my exercises I’ve got a basketball I just play with myself in the garage just trying to use my left hand [the affected limb] back and forth and just, like, do it over and over again…as long as I can tolerate it.
	Simplicity of set-up and operation	Context: suggestions for developers of game-based rehabilitation tools.
		Adult participant: Just to make it simple
		Adult participant: …we need things that are very plain, very simple because computers I mean he [his son] had it from kindergarten on. It’s so different for all of us right?

**Table 3:** Summary of main barriers to use and main enablers to therapy for pediatric focus group.

Features		Representative quotes^a^
**Main barriers to use**		
	Privacy and online safety	Context: follow-up conversation between parent and moderator
		CPP:…He trusts anyone, strangers, so I don’t feel comfortable with opening up a Facebook account for him
	Space requirement	Context: video games that support bilateral rehabilitation.
		CPP:…we don’t have the room in our house to accommodate all of that gross movement
**Main enablers to therapy**		
	Video games with a storyline	Context: what participants enjoy about the video games that they currently play.
		PP: I like it when you have like you make up like a pod then you get to like make your own city and get like troops…And you get to take over buildings and people and build a community.
	Incorporation of both the unaffected and affected limb	Context: potential issues for video games developed for home-based rehabilitation.
		PP:…you can’t use your hand that works perfectly, you have to control the guy with your affected and…that would be sooo boring
	Creates opportunities to connect with others	Context: what video games have to offer beyond rehabilitation
		PP: You know, if it would mean interacting with others kids who also have the same challenges, I think that would be pretty cool…someone that understands and gets it.
	Gaming for therapy vs gaming for leisure	Context: whether video games could motivate pediatric clients to adhere to their rehabilitation programs.
		PP: And if you want to play the therapy games you can play those therapy games, if you want to you can play your own kind of games

^a^PP: pediatric participant, CPP: caregiver of pediatric participant.

### Theme 1: Clients’ Use of Technology in Everyday Life and Rehabilitation

#### Technology and Home Use

When asked about the types of technology used within their home, participant responses were quite diverse. Adults and children differed in the types of technology they used. The adult participants reported regularly making use of mobile phones, Apple’s iPad, Facebook, email, and the Internet. In contrast, pediatric participants regularly used videogame consoles, such as Microsoft’s Xbox and Kinect, Sony’s PlayStation 2 and Move, as well as the Nintendo Wii. For the adult participants, the primary purpose for technology use was to acquire knowledge and information. One adult participant stated that, “you go to the doctor and he tells you stuff and you come home and look it up and you really know, you know” (Group 2, Line 580-581).

#### Technology for Social Interaction

Mobile phones, email, and Skype, which allow users to communicate by voice, video, and instant messaging over the Internet, were identified by adult participants as convenient ways to keep in touch with friends and family. Many of the adult participants reported that their use of technology was motivated by the opportunity to socialize and engage with others. When one adult participant was probed about her gaming experience, she responded that, “I’ve got two granddaughters so whatever they play, I play” (Group 2, Line 1129).

#### Technology for Entertainment

The pediatric participants’ current use of technology was primarily to play games for purely entertainment purposes rather than for social interaction. One parent reported that their child, “used a computer a lot at home for games” (Group 1, Line 8), but that they restricted the number of hours of play. Despite the popularity of social networking (eg, Facebook), the pediatric participants did not report the use of technology for the purpose of socializing with friends from home. Most pediatric participants were too young to be legally permitted to create a social networking account (eg, Facebook requires a minimum age of 13 years). Privacy and personal safety concerns were identified as barriers by caregivers (as discussed in Privacy Management and Personal Safety).

#### Therapeutic Use of Technology

All participants reported limited exposure to technology for rehabilitation. Discussion with the pediatric participants revealed that when technology was used during clinic visits, it was used by the clinician to motivate or to reduce boredom during therapy sessions. One parent reported that when their child performed the Superman pose (a floor exercise used in rehabilitation to strengthen back muscles whereby the child lies prone with back and arms extended, a position that resembles “Superman” flying, and one that is held for a short period of time), the physiotherapist placed an Apple iPad in front of their child. According to this parent, “our physio will actually put him in a Superman swing which, you know, so he’s on his hands and he’ll put an iPad in front of him…so then he has to weight bear on one hand and use the other one to play Ninja or whatever” (Group 1, Line 206-210). Technology was also used to break up the monotony of performing repetitive movement exercises and to re-engage the child. Another parent stated that it is, “often very motivating when, when they’re tired, and it’s like, okay, I don’t want to go chase that ball anymore but I can, I’ll play those video games for a while” (Group 1, Line 214-216).

### Theme 2: Barriers to Use

#### Privacy Management and Personal Safety

Security and privacy were primary concerns for both groups of participants when asked about combining social networking websites with home-based rehabilitation technology; the nature of these concerns was unique for each group. The parents of pediatric participants voiced that their major concern was the safety of social networking websites, especially if their child had any cognitive impairments. These impairments meant that their child might have difficulty creating appropriate boundaries, making them a target for predators. “He trusts anyone, strangers, so I don’t feel comfortable with opening up a Facebook account for him” (Group 1: follow-up conversation between parent and moderator). In contrast, the adult participants were apprehensive about public access to content available through personal profile page. According to the adult participants, the very nature of social networking encourages its users to reveal personal information. One adult participant stated, “I’m registered for Facebook, I don’t use it, I don’t, I don’t like to have everybody know my business” (Group 2, Line 550-551). They expressed that they did not understand why people choose to post family affairs on social networking websites. One adult participant asserted, “you’re private. You don’t put your stuff, your dirty laundry, out to dry” (Group 2, Line 565). In spite of these concerns, there seemed to be a general consensus among the parents of the pediatric and the adult participants that online games could be an innovative means of motivating clients to practice their therapeutic exercises. However, integrating it with social networking websites, such as Facebook, seemed to dissuade potential acceptance of such rehabilitation programs.

#### Cost

A potentially limiting factor identified by all the adult participants was the cost of the equipment for the proposed home-based rehabilitation system. The majority of the adult participants expressed that their decision to invest in technologies, such as robotics, would be largely dependent on the financial commitment they would have to make and the support or lack thereof that they might receive from government or other funding agencies. In contrast, the parents of the pediatric participants did not identify cost as a factor that would influence their use of a home-based rehabilitation device.

There was a general consensus among the adult participants that the government’s efforts to reduce health care costs by terminating payment for stroke rehabilitation before they had reached maximum recovery were very frustrating for them. This topic led to a candid discussion about the lack of government subsidy/support for rehabilitative therapy and the adult participants’ worry for those who could not afford the planned development of social gaming programs for home-based rehabilitation. There was also concern about equity of access to these new technologies, with one adult participant expressing that, “for people without money I don’t think that’s fair” (Group 2, Line 627-628).

#### Assurance of Therapeutic Improvement

To be convinced to use robotic technology and social gaming programs for rehabilitation, the adult participants wanted assurance that motor function would improve. One adult participant indicated, “We don’t have time, but if you said this is going to help you then we would do it…so you have to say I’m going to do this every day for 15 minutes, say, or whatever. And if it works and someone like me, you see a difference, well it spurs you on, right?” (Group 2, Line 1463-1466). When the moderator followed up asking, “if you see a difference with a therapy, it will keep you going?” (Moderator with Group 2, Line 1468), one of them replied, “Yes. Exactly” (Group 2, Line 1470).

#### Familiarity With Technology

Some adult participants expressed that their age stopped them from reaping the full benefits of video games and robotics technology aimed at motivating users to practice their daily therapy exercises. However, consistent with the previous theme, other adult participants expressed their willingness to adopt new technologies, especially if technologies were shown to be effective, for example, “If it’s going to help me, I’ll do it” (Group 2, Line 1204-1207). The adult participants reported that another major barrier to readily accepting a home-based robotic exercise program was their general lack of familiarity with social gaming programs and the time they would need to invest to learn about them. Many of the older adult participants expressed that, unlike the younger generation that had grown up with the Internet, they were less comfortable going “online”. They shared that they preferred to “live” their life rather than staying indoors playing “video games”. One adult participant stated, “You have to say, okay, this is my therapy. I think a lot of things about computers, I think a lot of the things, we’re not, we’re too busy living” (Group 2, Line 1458-1459). The adult participants did acknowledge that stroke also affects younger individuals, “I know there’s a lot of young people have strokes but most of us are older and, like, at this time computers are difficult for us” (Group 2, Line 1761-1762). They expressed that this type of rehabilitation technology would probably be valuable for younger people who had suffered a stroke. Despite their initial apprehension, the adult participants agreed that if the benefits of technology intervention for home-based rehabilitation could be demonstrated, they would be willing to invest both the time and the energy into utilizing it.

In contrast, pediatric participants were more uniform in their understanding of robotic technology, therapy games, and social media platforms. When asked about their comfort with technology, one pediatric participant claimed, “No, it’s pretty much, if you want to use it, then you have to get up and use it, figure it out” (Group 1, Line 1224). Another pediatric participant asserted, “I figured a lot of it out on my own” (Group 1, Line 908).

#### Space

Availability of space to play the games was also raised as an issue by the parents of the pediatric participants. The amount of room that would be necessary to practice the gross movements that the therapeutic exercises sometimes entail was identified as being potentially problematic. One parent stated, “we don’t have the room in our house to accommodate all that gross movement” (Group 1, Line 1262-1263).

### Theme 3: Enablers to Therapy

#### Overview

Enablers to therapy fell into a variety of subthemes that could be loosely grouped as potential motivators and desirable features for future therapy. Motivation and desirable features are intertwined (eg, desirable features would be motivating). Thus, the subthemes discussed below can be interpreted as motivators in current games, therapy, or technology and would therefore be desirable features for future therapies. All participants described mechanisms that would enhance their motivation and engage them in home-based rehabilitation therapies in which video games were integrated. Both the pediatric and the adult participants stressed the importance of having games that were both entertaining and enjoyable. The following subthemes were identified.

#### Going Beyond Clinically Prescribed Exercises

The adult participants stressed the need for video games that encourage diverse movement-based exercises from typical home-based exercises prescribed by clinicians (eg, towel rolling exercises) that tend to be the repetitive in nature. One adult participant stated, “it’s so boring to sit there and roll a towel up” (Group 2, Line 1405-1406). When the possibility of using a robotic device in therapy was raised, one of the adult participants responded, “So I think that would be a good idea because it’s hard finding things to do here I think, you know, that’s the problem with therapy, it’s so boring” (Group 2, Line 595-596).

#### Seeking “Deeper Stories” to Motivate

The pediatric participants were very clear about their desire for video games that were unique from those that social networking websites, such as Facebook, had to offer (eg, Candy Crush). There was a strong voice for video games that required “real time strategy” and offered “deeper stories”. While video games with esthetically pleasing graphics were an important consideration, these participants were also seeking a storyline, not just a game to play. Games previously played by this group that encouraged them to think and made them an active participant were exciting for them. “I like it when you have, like you make up like a pod, then you get to like make your own city and get like troops…And you get to take over buildings and people and build a community” (Group 2, Line 1052-1053). A few participants expressed their desire for games that would provide them with the opportunity to add to their gaming experience. Furthermore, these games should be multiplayer; however, participants were not stringent on whether these other “players” had to be family members, friends, or random opponents found online. They did like the idea of playing therapy games with others who had similar physical challenges.

#### Desire for Game-Based Therapy to Generate Results

The stance that the adult participants took regarding the entire rehabilitation process also distinguished them from the pediatric participants. They understood that structured exercises and the daily use of their affected limb were essential to achieve maximal functional gains. They also expressed their desire for the ability to adjust their therapy according to their individual needs—provide resistance and/or assistance, different motions, and to practice functional movements rather than single simple joint movements. According to the adult participants, game-based therapy should be competitive, challenging, and encourage the use of their affected limb. They recognized that to regain any degree of function of their affected limb would require intensive repetition of movement. They also accepted that they were accountable for continuing to perform their exercise regimen at home if they wished to make any significant progress toward their therapeutic goals. Finally, they expressed their willingness to put in the time and effort in order to see results. “In my exercises I’ve got a basketball I just play with myself in the garage just trying to use my left hand [the affected limb] back and forth and just, like, do it over and over again…as long as I can tolerate it” (Group 2, Line 1654-1655).

#### Need for Games to Incorporate the Unaffected Limb

The pediatric participants had a considerably different outlook on challenging themselves to actively engage their affected limb. They were firm in their appeal that video games be designed in such a way that they were not limited to using only their affected limb. Instead, they wanted video games that allowed them to utilize their entire body. One pediatric participant expressed his frustration when playing a video game that forced him to use only his affected limb, “you can’t use your hand that works perfectly, you have to control the guy with your affected and…that would be sooo boring” (Group 1, Line 1443-1444). When it came down to the aim of rehabilitation, the pediatric participants, unlike the adult participants, appeared much less concerned with long-term outcome. Their focus centered on the esthetic experience, enjoyment of gameplay, as well as, games that were not restricted to their affected limb only.

The parents of the pediatric participants expanded on the comments made by their children expressing the need to find ways to motivate their children to practice their exercises outside of the clinical setting to maximize functional ability. They described how any activity that engaged their child to use their affected limb without explicit instruction to do so was welcomed and encouraged: “a little girl taught him how to play a song on the piano, and he’s using his left hand [the affected limb] right now but he’s really into it” (Group 1, Line 355-357).

#### Technology as a Motivational Therapy Tool

Technology was identified by parents as positively tapping into their child’s motivation to comply with their exercise programs. The most challenging aspect for these parents was knowing that repetitive practice was necessary and getting their child to engage in practice regularly. They acknowledged that technology influenced their child’s intrinsic motivation and made exercising more enjoyable:

I thought it was really good. I think that especially, I can only speak to ReJoyce, but you could get him to do so many repetitions, whereas to do here you might get a child to shut down. They shut down. Whereas when you use the technology, they don’t even realize that they are doing the repetitions.

Group 1, Line 1262-1263

ReJoyce is a commercial therapy device for use in either a clinic or a client’s home by Rehabtronics Inc., Edmonton, Canada.

#### Opportunities to Interact With Peers

The idea of introducing technology to rehabilitation therapy that could be carried out at home was well received by the pediatric participants. Further probing by the moderator revealed that they were motivated by the prospects of connecting with others (ie, peers as well as other children with CP) in multiplayer online video games. There was a general sense that these particular pediatric participants had a harder time interacting with their schoolmates in physical games and activities because of their physical limitations. For them, video games offered a medium that leveled the playing field; they felt they could be equal to their typically developing counterparts. These children also acknowledged that playing with children with similar disabilities (CP) would be exciting, “You know, if it would mean interacting with others kids who also have the same challenges, I think that would be pretty cool…someone that understands and gets it” (Group 1, Line 56-58).

#### Need for Simplicity

The adult participants identified simplicity as essential when designing technology for home-based rehabilitation purposes. Their adoption of rehabilitation technology would depend on ease of use. It was important that the amount of time invested in setting the system up was minimal and that the games were simple to initiate, understand, and play. “Clear, clear directions and using words that you all know” (Group 2, Line 1757).

Once more, the adult participants felt their unfamiliarity with technology was a disadvantage. As a consequence, they anticipated that it would take them longer to learn to use the technology, and this was time that they did not want to waste. Time was very valuable to the adult participants. One adult participant expressed, “Just make it simple” (Group 2, Line 1732), while another adult participant explained that, “we need things that are very plain, very simple because computers, I mean he [his son] had it from kindergarten on. It’s so different for all of us, right?” (Group 2, Line 1738-1739).

#### Distinction Between Gaming for Therapy and Gaming for Leisure

An issue that was strongly and frequently vocalized throughout the pediatric focus group discussion was the need to develop games that are dedicated solely to supporting rehabilitation. If the purposes of these games were to enhance rehabilitation therapy, motivate clients, and promote adherence, then they needed to be unique from those that were played during their free time. One pediatric participant stated*,* “I think it would actually be very useful because you can have certain games for therapy and certain games for your own free time. And if you want to play the therapy games, you can play your therapy games, and if you want to you can play your own kind of games” (Group 1, Line 1128-1130). Another expressed, “if you have your video games to motivate your therapy then you can’t play with them much on your own” (Group 1, Line 1117-1118). When the moderator probed this statement by asking, “Do you think that it would stop you from wanting to play them on your own?” (Group 1, Line 1120), the participant responded, “Yeah…because then you’d have to do therapy more often” (Group 1, Line 1122, 1126). The concern that participants expressed appeared to be that game-based therapy would take away from gameplay for pleasure. Furthermore, they were uneasy about being monitored while playing therapeutic video games either in the clinical setting or at home by their parents, “people actually watch what you’re doing, that freaks me out because they can watch you, it’s kind of creepy like they’re basically watching you” (Group 1, Line 974-976). They were concerned that they would be subjected to continual scrutiny not only by their therapist but also by their parents.

## Discussion

### Principal Results

This study was undertaken to examine how adult and pediatric clients with upper-limb hemiparesis were using technology in everyday life and rehabilitation. It also aimed to explore the perceived benefits and barriers to incorporating technology for upper-limb rehabilitation in two different age groups with similar etiology. These findings provide a descriptive perspective of the generational differences in technology use and highlight the need for well-designed systems that are highly user-specific. Three major themes emerged in this study and were central to understanding the participants’ perceptions of technology and its potential use for rehabilitation: (1) clients’ use of technology in everyday life and rehabilitation, (2) barriers to use, and (3) enablers to therapy. It was clear from the results that all participants had some degree of experience with technology: the adult group using technologies predominantly for communication and information gathering, while the pediatric group used technology for primarily entertainment purposes. This sample of participants reported minimal exposure to technology for therapeutic purposes, but all acknowledged the potential benefits of technology in providing motivation and interest for the performance of repetitive task practice. Determinants of adoption and use of therapeutic technologies for upper-limb rehabilitation differed between age groups. The adult participants appeared to balance benefits in terms of effectiveness, capacity to provide feedback, customization to their specific requirements and ability to offer differing options to current home exercise programs against the monetary costs, and efforts involved in adoption and use. The pediatric participants reported that they value the quality of entertainment and opportunities to interact with peers. They also expressed maximization of opportunities for success in the gameplay over therapeutic benefit and the desire for a distinction between gaming for therapeutic versus leisure purposes. Privacy and personal safety concerns were raised by both groups in those instances that social media would be incorporated to monitor progress into the therapeutic technology paradigm. The barriers and enablers to the adoption of therapeutic technologies differed between the user age groups. These findings may assist researchers in targeting the development and design of future technologies for therapeutic use in a home setting to these populations.

### Limitations

These focus group data are limited by demographics of the individuals participating. That is, the data do not necessarily represent a range of socioeconomic and cultural views in relation to this topic, and no socioeconomic data/cultural data were obtained from participants. Furthermore, the majority of the pediatric client group were male (6 male, 1 female) and there was a wide age range (6-16 years of age). Another drawback of our study is its small sample size; however, our findings should be viewed as exploratory, offering game developers insights from these two populations (children with ABI and adults post stroke).

All participants were from a public/private health care system in an urban area where long-term care is capped; however, they did have access to a number of resources and supports. Our results may have been different had we run our focus group in a rural community, as it is possible that the motivation to consider alternate means of undertaking upper-limb rehabilitation may be influenced by the availability of resources. Furthermore, all participants were volunteers who knew the general aim of the study. Self-selection on the part of the participants may have biased the results of the focus group.

### Comparison With Prior Work

The current uses and perceptions of technology reported in this study are consistent with trends previously published regarding the general population [26]. A recent study by Gell et al [27] examined technology use among older adults and found that as physical capacity decreased, so did usage; however, these results were also influenced by the type and degree of disability. Surprisingly, the participants in this study described minimal use of rehabilitative or gaming technologies in their rehabilitation, which contrasts considerably with some countries, such as Australia, where up to 76% of stroke rehabilitation facilities use commercial gaming systems such as the Nintendo Wii [28].

The determinants and modifiers to the adoption and use of rehabilitative technologies for upper-limb rehabilitation identified in this study are congruent with many of the constructs presented by Venkatesh et al [29] in their Unified Theory of Acceptance and Use of Technology. This theory proposed four categories of determinants for acceptance and use: (1) “performance expectancy” related to identified potential benefits of the technology (eg, effectiveness, quality of experience), (2) “effort expectancy”, or how the effectiveness is balanced against the effort and costs, (3) “social influence” (eg, image, social factors, and norms), which is linked to the degree that others’ expectations influence a user’s adoption of technology, and (4) the “facilitating conditions” (eg, simplicity, training, perceived behavioral control) that support the use of the system. Key modifiers to behavioral intention were the gender, age, and experience of the user and the extent to which use of the technologies was voluntary.

The information provided by participants in this study suggests that age may be an important factor in the determinants of technology adoption and use. Performance expectancy elements differed between age groups. The adult participants placed greater weight on effort expectancy constructs, whereas the pediatric group appears to place greater emphasis on social determinants. This was closely linked to the performance expectancy constructs. Designers should be cognizant of a balance between demanding sufficient practice and allowing pediatric users the opportunities to play using their unaffected limb (or perhaps integrating both through bimanual controls) and to make social connections with others. The pediatric participants saw the technologies as providing a medium where they could engage with and perform equally with typically developing peers.

The modifiers identified by the groups also differed. While not specifically evaluated, the adult participants identified user experience as a potentially influential modifier; however, they suggested facilitators that could counter this modifier, including simplicity of use. Applications should be easy for users to set up, and the games should be relatively intuitive and easy to learn in efforts to minimize inexperienced users’ anxiety, increase the likelihood of adoption, and increase the likelihood of protracted use [30,31]. An influential modifier for pediatric participants was perceived behavior control related to differentiating gaming for therapy from leisure gaming time. There was also concern regarding scrutiny by therapists and parents during gameplay.

### Conclusions

The application of robotics combined with gaming technology is becoming an increasingly popular means of supporting upper-limb rehabilitation. When designing appropriate devices/systems, it is not enough to simply focus on functionality and cost. Consideration needs to be given to their appropriateness and acceptability to their users, which makes user involvement in research invaluable and essential [32-34]. Both the pediatric and the adult participants were open to the integration of technology into rehabilitation; nevertheless, some differences became evident upon further investigation. The adult participants were more pragmatically motivated by potential recovery gains. The younger participants were more intrinsically motivated by access to play games, especially the potential to use games as a platform for socializing and competing with their typically developing peers. Based on the feedback from the study’s participants, a successful gaming system should consider the following: incur low cost, demonstrate improved recovery, be simple to operate, be space-efficient, prescribe unique exercises, offer challenging and motivating games, incorporate the unaffected limb(s), create social connections, and demonstrate a clear distinction between gaming for therapy and for leisure. To understand more clearly the needs of potential users, directions for future research should include clinicians’ perspectives of technology and rehabilitation [20], and the development of rehabilitation robotics and refinement to existing prototypes based on the information gathered in this study.

## Multimedia Appendix 1

Focus group questions.

## Abbreviations

ABI: acquired brain injury
CP: cerebral palsy
CPP: caregiver of pediatric participant
PP: pediatric participant
