# Supplementary material for: Perceptions of Technology and Its Use for Therapeutic Application for Individuals With Hemiparesis: Findings From Adult and Pediatric Focus Groups
Source: JMIR Rehabil Assist Technol. 2015 Feb 10;2(1):e1. doi: 10.2196/rehab.3484 (PMC5454546; doi:10.2196/rehab.3484)
Supplement: Supplementary file 1 [file rehab_v2i1e1_app1.pdf]

Multimedia Appendix 1. Sample focus group questions.

1. Can you please tell us about the kinds of arm and hand activities that your Occupational or Physical Therapists (OT's or PTs) ask you to do at home?

- Probes:
    - How many types of exercises/activities?
    - What kinds?
    - Range of motion exercises?
    - Exercises/Activities to make your hand and arm stronger?
    - Games?
    - How often do you do them?
    - Do you use equipment?
    - Do you need help to do the exercises?
    - What kinds of activities do you do in therapy?

2. Can you please tell us about the kinds of splints and other devices that you use at home?

- Probes:
    - What are they used for? Do you wear them at rest (when you are not doing anything)?
    - Do you use them to complete home exercise programs?
    - How often do you wear them?
    - When do you wear them? (At night or during the day)?
    - Do you/your child need help putting them on?

3. Do you use technology, such as: social media, computerized programs or computer games at home? If so, what types of technology do you use?

- Probes:
    - Devices: iPads, desktops, iPhones, etc.
    - Are there certain types of technology you use more than others? (i.e. iPad/iPod touch)
    - Do you play games on their own or with others?
    - Systems: Wii, Kinect, etc.
    - Social media examples are: Facebook, Twitter, Linked-in, Google Plus, Skype, YouTube,
    - How many of your have a Facebook account or Google Plus or anything else?
    - Games: Super Mario, Wii fit, Angry Birds, Sudoku, Dance Dance Revolution, multiplayer games, Farmville etc.

4. How do you use them?

- Probes:
    - How often do you use the technology?
    - What are your favourite kinds? What makes these your favourite?
    - How do you value technology (and games/favourites) in your life?
    - What kind of assistance do you need to use them?

- What are some challenges for you to using them?
- What would make it easier for you to use social media, computerized programs or games at home?
- Are there other technologies that you know about or would like to know about?

5. Tell us what you think about using social media to help motivate you to practice your home exercise programs. (Remind them what social media is i.e. Twitter/Facebook/steam platform?)

- Probes:
    - Could it be beneficial (useful)? If so, why? If not, why not?
    - What might make it hard to use social media?
    - What would you like the research team to include when they design a tool to help you play with social media?
    - If you were able to ask the team to design a system that you could use, what kinds of things would you like to see in the system?
    - What would help you play with it?
    - What features of social media would be important for the research team to avoid in the design of a rehabilitation tool?
    - Do you have ideas about how to find solutions for therapists to integrate social media use with clients?

6. Tell us what you think about using a robot to help motivate you to practice their home exercise programs.

- Probes:
    - Could it be beneficial/useful? If so, why? If not, why not?
    - What might make it hard to use a robot?
    - What features of robotics would be important for the research team to incorporate in the design?
    - What features of robotics would be important for the research team to avoid incorporating into the design?
    - Anything else you wish to add?
